# Bacteria under SOS evolve anticancer phenotypes

**DOI:** 10.1186/1750-9378-5-3

**Published:** 2010-02-05

**Authors:** Shatha F Dallo, Tao Weitao

**Affiliations:** 1 Biology Department, the University of Texas at San Antonio, One UTSA Circle, San Antonio, Texas 78249-0662, USA

## Abstract

**Background:**

The anticancer drugs, such as DNA replication inhibitors, stimulate bacterial adhesion and induce the bacterial SOS response. As a variety of bacterial mutants can be generated during SOS, novel phenotypes are likely to be selected under the drug pressure.

**Presentation of the hypothesis:**

Bacteria growing with cancer cells in the presence of the replication inhibitors undergo the SOS response and evolve advantageous phenotypes for the bacteria to invade the cancer cells in order to evade the drug attack. This hypothesis predicts that bacteria produce the proteins that mediate bacterial capture and invasion of cancer cells--the advantageous phenotypes. Generation of the phenotypes may be facilitated during the SOS response induced by anticancer drugs.

**Testing the hypothesis:**

Experimental design: 1) Examine attachment and invasion of bacterium *Pseudomonas aeruginosa *and the SOS mutant control to cancer cells in the presence of the anticancer drugs that inhibit DNA replication enzymes and trigger the SOS response. 2) Reveal the bacterial proteins that exhibit changes in expression. 3) Identify the genes encoding cancer adhesion and invasion. 4) Construct the mutants for the genes, clone and express these genes. 5) Examine the bacterial capture and invasion of cancer cells in contrast to non-cancer control.

Expected results: 1) The bacterial proteins will be differentially induced during bacteria-cancer interaction under the SOS response to the anticancer drugs. 2) Knocking out the bacterial cancer-adhesion-invasion genes will disrupt the adhesion-invasion phenotypes of the bacteria. 3) Expressing these genes will direct the bacterial capture and invasion of cancer cells.

**Implications of the hypothesis:**

Bacteria can evolve anticancer phenotypes targeting metastatic cells. If this hypothesis is true, the outcomes will contribute to development of a novel bacterial anti-metastasis regimen.

## Background

Attempts with live bacteria to control cancer progression were tried over a century ago [1,2]. Although undesired infections raised a concern, creative hypotheses and progress have sparkled. As reviewed by Chakrabarty [3], antitumor treatment with *Clostridium novyi *was proposed, based on propensity of the anaerobe to grow in anaerobic core of the tumors and to deprive tumors of oxygen and essential nutrients. *Salmonella*, a facultative anaerobe, was also found to have tumor propensity that appears to be encoded by the pathogenicity island. Furthermore, bacteria could be engineered for selective destruction of tumors and for bacterial gene-directed prodrug therapy; in fact, such bacteria appeared to kill tumors selectively but not the normal tissue [4]. While these data support the notion of bacterial tropism and cancer killing, it remains unclear how they are developed and what evolutionary relationship of bacteria is with cancer.

We previously proposed the analogy of bacterial lifestyle to cancer cell behaviors, projecting the evolutionary relationship [5,6]. The shared features are reflected by observations that bacteria and cancer cells respond similarly to such anticancer drugs as DNA replication inhibitors [5]. These common lifestyles imply that they may compete with each other under certain conditions [5,6]. Bacteria growing under competition and drug influence are highly likely to evolve new phenotypes against cancer.

**Figure 1:**
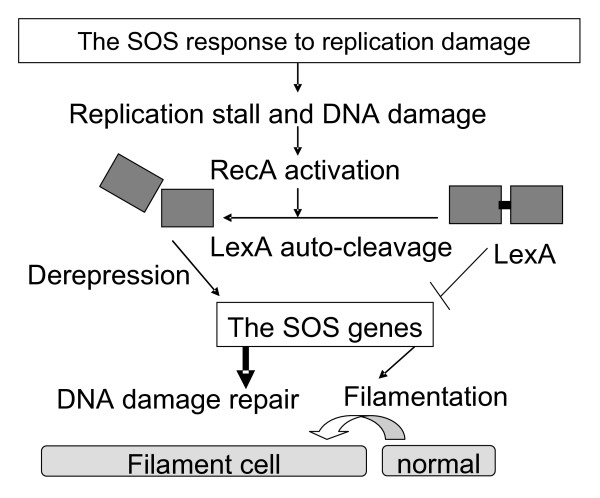
**The SOS response**. LexA and RecA control the SOS genes that encode functions required for DNA damage repair. LexA represses these genes. DNA damage activates RecA to stimulate autocatalytic cleavage of LexA so that the SOS genes are derepressed and expressed for repair. Cell division is inhibited and delayed resulting filamentation to allow repair before cell division.

Replication inhibitors also induce the SOS response [7] during which generation of new phenotype may be facilitated. SOS is a transcriptional response, in which at least 40 SOS genes in *E. coli *[8-10] and 15 in *P. aeruginosa *[11] are induced through interplay of the SOS regulators LexA and RecA (Fig. 1). In the presence of single-stranded DNAs that are generated during replication inhibition, RecA coprotease senses the signals and binds to the single-stranded DNA to assume an active conformation [12] and to stimulate auto-cleavage of LexA [13]. Consequently, LexA repression of the SOS genes is prevented by this cleavage leading to a global induction of the SOS response.

**Figure 2:**
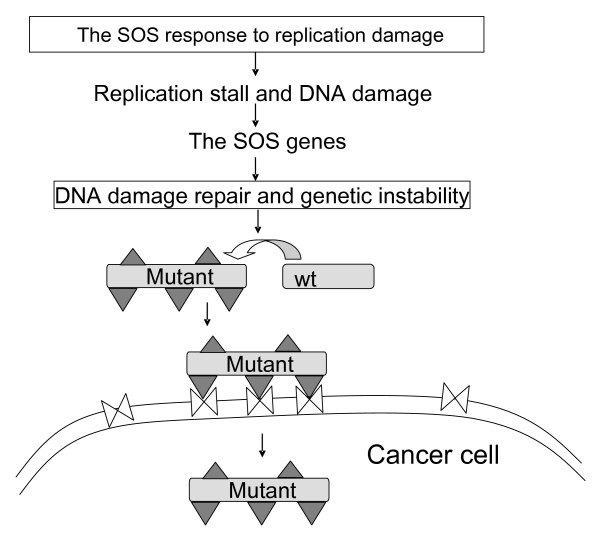
**The hypothesis of bacterial cancer capture-invasion phenotypes**. The bacterial SOS response is triggered by DNA damage caused by treatment with replication inhibition anticancer drugs. If damage is so extensive that the cells cannot directly repair, the lesions of damage can be bypassed, leading to mutagenesis and genetic instability. A variety of bacterial mutants can be generated consequently and selected for adhesion to cancer cells and invade them to evade the drug attack. The mutants produce proteins (triangles) that recognize the cancer cells surface (double triangles) and mediate bacterial adhesion to the cancer cells.

These SOS gene products are involved in cytogenesis, DNA recombination, DNA replication, DNA damage repair, and segregation of chromosomes during cell division [14,15]. For instance, the SOS gene, *sulA*, is induced to inhibit and delay cell division transiently leading to cell filamentation (Fig. 1) until DNA damage is ameliorated. The SOS-controlled *umu *operon is involved in the error-prone translesion DNA synthesis [16]. If damage is so extensive that it cannot be directly repaired, the lesions of damage can be bypassed by the translesion synthesis with aid of the *umu *encoded proteins [17], leading to mutagenesis and genetic instability. A variety of bacterial mutants can be generated consequently. If bacteria grow with cancer and anticancer drugs, pools of these bacterial mutants are, in fact, selected for new phenotypes (Fig. 2).

This article aims to present a creative hypothesis as below. This hypothesis predicts that bacteria can evolve the cancer adhesion-invasion phenotypes, to challenge the limitation of anticancer treatment arising from bacterial natural propensity to cancer. The outcomes should help develop novel bacterial anticancer regimens to deal with the safety and specificity issues poised over a century ago.

## Presentation of the hypothesis

Our hypothesis states that bacteria, growing with cancer cells and replication inhibition drugs, evolve advantageous phenotypes. This hypothesis suggests that treated with the drugs, bacteria can be induced to adhere to and to invade cancer cells so that bacteria survive the drug attack. These features are defined as the cancer adhesion-invasion phenotypes. Obviously, our hypothesis is not based on the bacterial natural antitumor propensity but on the SOS-induced molecular evolution of new phenotypes. This hypothesis will be tested with *P. aeruginosa *as a starter. While its ecological niche may not be necessarily tumor, this bacterium could attach to and penetrated human lung epithelial cells derived from a human bronchus alveolar carcinoma [18]. These antitumor activities may be mediated by bacterial proteins; in fact, *P. aeruginosa *does have such an antitumor potential since it has genes encoding antitumor proteins. Azurin is a periplasmic antitumor protein in *P. aeruginosa *(reviewed in [19]). Release of Azurin depends on contact with cancer cells, and Azurin targets preferentially cancer cells but marginally normal cells [20]. Additionally, Laz and Pa-CARD displays cytotoxic activity against leukemia cells [21]. Our hypothesis suggests that such proteins and new candidates would emerge when bacteria undergoing the anticancer drugs-induced SOS mutagenesis interact with cancer cells.

Furthermore, bacterial adhesion to cancer may be induced as we proposed previously [6]. *P. aeruginosa *can be induced to attach to abiotic surface and can form biofilms in response to hydroxyurea [5,22]. While historically it is an antiproliferative drug for tumor treatment [5,22], hydroxyurea is a replication inhibitor targeting at ribonucleotide reductases that are a good anticancer target [23]. This drug inhibits growth of proliferating planktonic bacterial cells but stimulates bacterial adhesion [5,22], likely to cancer. Such replication inhibitors induce the bacterial SOS response (Fig. 1) [7] during which generation of the advantageous phenotypes may be facilitated (Fig. 2). For instance, error prone DNA replication generates mutagenesis and genetic instability during SOS, yielding a variety of bacterial mutants. Since bacterial entry into cancer cells can evade the drug attack, these mutants can be selected for cancer invasion for bacterial survival.

## Testing the hypothesis

### Experimental design

**Figure 3:**
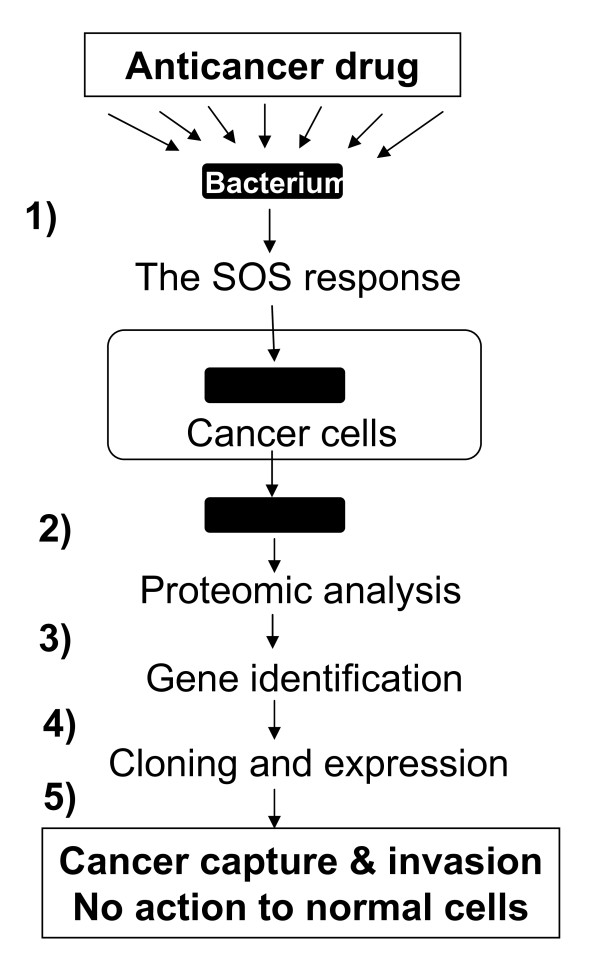
**Experimental design**. 1) Examine bacterial attachment to and invasion into cancer cells in the presence of the anticancer drug that induces the SOS response. 2) Use proteomic analysis to reveal the bacterial proteins that exhibit changes in expression. 3) Identify the genes encoding cancer adhesion and invasion. 4) Construct the mutants for the genes, clone and express these genes. And 5) examine the bacterial capture and invasion of cancer cells in contrast to non-cancer control.

To test this hypothesis, we will first examine attachment of *P. aeruginosa *to cancer cells and cancer invasion (Fig. 3). We will use the *recA *mutant as a bacterial SOS control because RecA initiates SOS that may facilitate development of the adhesion-invasion phenotypes. We will incubate these bacterial cells with cancer cells in the presence of the anticancer drugs that inhibit DNA replication enzymes including DNA polymerases [24], DNA helicases [25], ribonucleotide reductases [23], and topoisomerases [26]. We will harvest the invaded bacteria from the cancer cells. Second, we will use proteomic analysis to reveal proteins that exhibit distinct changes in expression in the bacterial cells that adhere to and invade into cancer cells. Lastly, according to the proteomic results, we will identify the mutated genes encoding cancer adhesion and invasion. We will construct the deletion mutants by deletion-insertion of the genes encoding the induced proteins. We will test the mutants for cancer adhesion and invasion. We will over-produce these proteins and examine adhesion-invasion phenotypes against the cancer cells and the non-cancer control.

### Bacterial proteins differentially induced during bacteria-cancer interaction

Bacteria under the anticancer drug-induced SOS are expected to produce proteins that mediate the cancer adhesion-invasion phenotypes (Figs. 2 and 3). The protein induction is generally reflected by alterations in the intensities of the 2-D gel protein spots from the invaded and non-invaded bacteria, with reference to the SOS controls. Interacting with cancer cells, the bacteria are likely to evolve a pattern of surface proteins suitable for bacterial adhesion to and invasion of cancer cells (Fig. 2). The pattern is likely to be unique to the cancer adhesion-invasion phenotypes as compared with that of the SOS and non-invaded controls. These proteins are expected to be deficient in the *recA *mutant, in which SOS is precluded. They are unlikely to appear on the non-invaded bacterial control.

### Identification of bacterial genes encoding cancer adhesion and invasion

It is plausible that knocking out the bacterial cancer-adhesion-invasion genes would disrupt the adhesion-invasion phenotypes. If the proteomic analysis indicates increases in the levels of the proteins from the bacteria with the adhesion-invasion phenotypes, mutations with inactivation of the genes encoding these proteins will render the mutant bacteria unable to attach to and invade the cancer cells. However, over-production of these proteins is expected to enhance bacterial capture and invasion of the cancer cells but not the non-cancer control. Then, it can be concluded that these genes are required for the cancer adhesion-invasion phenotypes.

Collectively, the high resolution of the 2-D based proteomic approach will allow us to identify the patterns of the cancer-inducible surface proteins on bacteria so that the encoding genes can be identified. These genes can be cloned and expressed in non-pathogenic bacteria that are safe to the hosts but selectively lethal to cancer cells.

## Implications of the hypothesis

Our hypothesis regarding evolution of bacterial anticancer phenotypes implies that bacteria can evolve cancer cell-specific phenotypes when growing with cancer cells of certain types, for instance, the metastatic cells. Metastasis is a process in which cancer cells migrate to distant sites and adapt to the tissue microenvironment from the primary cancer and thus becomes the major cause of deaths in cancer patients. While metastasis can be impaired by the antagonist bacterial biofilms [6] or by the bacterial proteins [19-21] during treatment with the anticancer drugs as proposed previously [6], the underlying mechanism for bacterial recognition or tropism for cancer cells is not fully understood and thus addressed by this hypothesis. As bacterial tropism is still in its infancy, if this hypothesis is true, the outcomes may stimulate future research interest into development and evolution of the bacterial tropism for cancer cells, contributing to formulating novel bacterial anti-metastasis regimens.

## Competing interests

The authors declare that they have no competing interests.

## Authors' contributions

TW contributed to original conception. He designed and composed the hypothesis. SFD performed analysis and interpretation of data of the manuscript and revised it critically for important intellectual excitement.

## Authors' informations

Dr. Weitao who currently holds a faculty position at the University of Texas at San Antonio is an expert in areas of microbiology and molecular biology. Dr. Dallo is an expert in microbiology and immunology in Dr. Weitao's group. This group is interested in breaking ground, creating new hypotheses and theories to tackle the unbeatable challenges, such as cancer.

## References

[B1] Coley WB The treatment of malignant tumours by repeated inoculations of erysipelas. With a report of ten original cases. 1893 Clin Orthop Relat Res 1991 262 3 11 1984929

[B2] Nauts HC Swift WE Coley BL The treatment of malignant tumours by bacterial toxins as developed by the late William B. Coley, MD, reviewed in the light of modern research Cancer Res 1946 6 205 216 21018724

[B3] Chakrabarty AM Microorganisms and Cancer: Quest for a Therapy J Bacteriol 2003 185 9 2683 2686 10.1128/JB.185.9.2683-2686.2003 12700245 PMC154404

[B4] Jain KK Use of bacteria as anticancer agents Expert Opinion on Biological Therapy 2001 1 2 291 300 10.1517/14712598.1.2.291 11727536

[B5] Weitao T Multicellularity of a unicellular organism in response to DNA replication stress Research in Microbiology 2009 160 1 87 88 10.1016/j.resmic.2008.09.010 18992808

[B6] Weitao T Bacteria form biofilms against cancer metastasis Medical Hypothesis 2009 72 4 477 478 10.1016/j.mehy.2008.11.012 19091481

[B7] Walker GC Mutagenesis and inducible responses to deoxyribonucleic acid damage in Escherichia coli Microbiol Mol Biol Rev 1984 48 1 60 93 10.1128/mr.48.1.60-93.1984 PMC373003 6371470

[B8] Fernandez de Henestrosa AR Ogi T Aoyagi S Chafin D Hayes JJ Ohmori H Woodgate R Identification of additional genes belonging to the LexA regulon in Escherichia coli Molecular Microbiology 2000 35 6 1560 1572 10.1046/j.1365-2958.2000.01826.x 10760155

[B9] Courcelle J Khodursky A Peter B Brown PO Hanawalt PC Comparative Gene Expression Profiles Following UV Exposure in Wild-Type and SOS-Deficient Escherichia coli Genetics 2001 158 1 41 64 11333217 10.1093/genetics/158.1.41 PMC1461638

[B10] Khil PP Camerini-Otero RD Over 1000 genes are involved in the DNA damage response of Escherichia coli Molecular Microbiology 2002 44 1 89 105 10.1046/j.1365-2958.2002.02878.x 11967071

[B11] Cirz RT O'Neill BM Hammond JA Head SR Romesberg FE Defining the Pseudomonas aeruginosa SOS Response and Its Role in the Global Response to the Antibiotic Ciprofloxacin J Bacteriol 2006 188 20 7101 7110 10.1128/JB.00807-06 17015649 PMC1636241

[B12] Sassanfar M Roberts JW Nature of the SOS-inducing signal in Escherichia coli: The involvement of DNA replication Journal of Molecular Biology 1990 212 1 79 96 10.1016/0022-2836(90)90306-7 2108251

[B13] Little JW Mechanism of specific LexA cleavage: autodigestion and the role of RecA coprotease Biochimie 1991 73 4 411 421 10.1016/0300-9084(91)90108-D 1911941

[B14] Cox MM A broadening view of recombinational DNA repair in bacteria Genes to Cells 1998 3 2 65 78 10.1046/j.1365-2443.1998.00175.x 9605402

[B15] Sherratt DJ Bacterial Chromosome Dynamics Science 2003 301 5634 780 785 10.1126/science.1084780 12907786

[B16] Kitagawa Y Akaboshi E Shinagawa H Horii T Ogawa H Kato T Structural Analysis of the umu Operon Required for Inducible Mutagenesis in Escherichia coli Proceedings of the National Academy of Sciences 1985 82 13 4336 4340 10.1073/pnas.82.13.4336 PMC390408 2989817

[B17] Bridges BA Woodgate R Mutagenic Repair in Escherichia coli: Products of the recA Gene and of the umuD and umuC Genes Act at Different Steps in UV-Induced Mutagenesis Proceedings of the National Academy of Sciences 1985 82 12 4193 4197 10.1073/pnas.82.12.4193 PMC397962 3889923

[B18] Carterson AJ Honer zu Bentrup K Ott CM Clarke MS Pierson DL Vanderburg CR Buchanan KL Nickerson CA Schurr MJ A549 Lung Epithelial Cells Grown as Three-Dimensional Aggregates: Alternative Tissue Culture Model for Pseudomonas aeruginosa Pathogenesis Infect Immun 2005 73 2 1129 1140 10.1128/IAI.73.2.1129-1140.2005 15664956 PMC547019

[B19] Mahfouz M Hashimoto W Das Gupta TK Chakrabarty AM Bacterial proteins and CpG-rich extrachromosomal DNA in potential cancer therapy Plasmid 2007 57 1 4 17 10.1016/j.plasmid.2006.11.001 17166586

[B20] Yamada T Fialho AM Punj V Bratescu L Gupta TKD Chakrabarty AM Internalization of bacterial redox protein azurin in mammalian cells: entry domain and specificity Cellular Microbiology 2005 7 10 1418 1431 10.1111/j.1462-5822.2005.00567.x 16153242

[B21] Kwan JM Fialho AM Kundu M Thomas J Hong CS Das Gupta TK Chakrabarty AM Bacterial proteins as potential drugs in the treatment of leukemia Leuk Res 2009 33 10 1392 1399 10.1016/j.leukres.2009.01.024 19250673

[B22] Gotoh H Zhang Y Dallo SF Hong S Kasaraneni N Weitao T Pseudomonas aeruginosa under DNA replication inhibition tends to form biofilms via Arr Research in Microbiology 2008 159 4 294 302 10.1016/j.resmic.2008.02.002 18434096

[B23] Shao J Zhou B Bernard Chu Yen Y Ribonucleotide Reductase Inhibitors and Future Drug Design Current Cancer Drug Targets 6 5 409 431 10.2174/156800906777723949 16918309

[B24] Berdis AJ DNA Polymerases as Therapeutic Targets Biochemistry 2008 47 32 8253 8260 10.1021/bi801179f 18642851 PMC2692436

[B25] Sharma S Doherty KM Brosh RM Jr DNA helicases as targets for anti-cancer drugs Curr Med Chem Anticancer Agents 2005 5 3 183 199 10.2174/1568011053765985 15992349

[B26] Teicher BA Next generation topoisomerase I inhibitors: Rationale and biomarker strategies Biochemical Pharmacology 2008 75 6 1262 1271 10.1016/j.bcp.2007.10.016 18061144

